# Super-additive associations between parity and education level on mortality from cardiovascular disease and other causes: the Japan Collaborative Cohort Study

**DOI:** 10.1186/s12905-022-01805-y

**Published:** 2022-07-06

**Authors:** Sumiyo Yasukawa, Eri Eguchi, Akiko Tamakoshi, Hiroyasu Iso, for JACC Study Group, Akiko Tamakoshi, Hiroyasu Iso, Mitsuru Mori, Yoshihiro Kaneko, Ichiro Tsuji, Yosikazu Nakamura, Kazumasa Yamagishi, Haruo Mikami, Michiko Kurosawa, Yoshiharu Hoshiyama, Naohito Tanabe, Koji Tamakoshi, Kenji Wakai, Masahiko Ando, Koji Suzuki, Shuji Hashimoto, Hiroshi Yatsuya, Shogo Kikuchi, Yasuhiko Wada, Satoe Okabayashi, Kotaro Ozasa, Kazuya Mikami, Kiyomi Sakata, Yoichi Kurozawa, Yoshihisa Fujino

**Affiliations:** 1 Department of Health Sciences, Institute of Academic and Research, Okayama University, 2-5-1 Shikata-cho, Kita-ku, Okayama, 700-8558 Japan; 2 Department of Epidemiology, School of Medicine, Fukushima Medical University, 1 Hikarigaoka, Fukushima, 960-1295 Japan; 3 Department of Public Health, Hokkaido University Faculty of Medicine, 15 Nishi 7, Kita-ku, Sapporo 060-8638 Japan; 4 Public Health, Department of Social Medicine, Osaka University Graduate School of Medicine, 2-2 Yamadaoka, Suita, Osaka 565-0871 Japan; 5 Hokkaido Chitose College of Rehabilitation, 2-10 Satomi, Chitose, Hokkaido 066-0055 Japan; 6 Japan Support Center for Suicide Countermeasures, 4-1-1 Ogawa-Higashi, Kodaira, Tokyo 187-8551 Japan; 7 Tohoku University Graduate School of Medicine, 2-1 Seiryo-machi, Aoba-ku, Sendai, Miyagi 980-8575 Japan; 8 Jichi Medical School, 3311-1 Yakushiji, Shimotsuke, Tochigi 329-0498 Japan; 9 Faculty of Medicine, University of Tsukuba, 1-1-1 Tennodai, Tsukuba, Ibaraki 305-8575 Japan; 10 Chiba Cancer Center Research Institute, 666-2 Nitona-cho, Chuo-ku, Chiba, Chiba 260-8717 Japan; 11 Juntendo University Faculty of Medicine, 2-1-1 Hongo, Bunkyo-ku, Tokyo 113-8421 Japan; 12 Yokohama Soei University, 1 Mihocho, Midori-ku, Yokohama, Kanagawa 226-0015 Japan; 13 University of Niigata Prefecture, 471 Ebigase, Higashi-ku, Niigata, Niigata 950-8680 Japan; 14 Nagoya University Graduate School of Health Sciences, 1-1-20 Daiko-Minami, Higashi-ku, Nagoya, Aichi 461-8673 Japan; 15 Nagoya University Graduate School of Medicine, 1-1-20 Daiko-Minami, Higashi-ku, Nagoya, Aichi 461-8673 Japan; 16 Nagoya University Hospital, 65 Tsurumaicho, Showa-ku, Nagoya, Aichi 466-8560 Japan; 17 Fujita Health University School of Health Sciences, 1-98 Dengakugakubo, Kutsukakecho, Toyoake, Aichi 470-1192 Japan; 18 Fujita Health University School of Medicine, 1-98 Dengakugakubo, Kutsukakecho, Toyoake, Aichi 470-1192 Japan; 19 Aichi Medical University School of Medicine, 2-10-31 Tsutsui, Higashi-ku, Nagoya, Aichi 461-8641 Japan; 20 Wakayama Prefecture Tanabe Public Health Center, 23-1 Asahigaoka, Tanabe, Wakayama 646-0027 Japan; 21 Kyoto University Health Service, Yoshida Hommachi, Sakyo-ku, Kyoto, Kyoto 606-8317 Japan; 22 Radiation Effects Research Foundation, 5-2 Hijiyama Koen, Minami-ku, Hiroshima, Hiroshima 732-0815 Japan; 23 Japanese Red Cross Kyoto Daiichi Hospital, 15-749 Hommachi, Higashiyama-ku, Kyoto, Kyoto 605-0981 Japan; 24 Iwate Medical University, 1-1-1, Idai-dori, Yahaba-cho, Shiwa-gun, Iwate 028-3694 Japan; 25 Faculty of Medicine, Tottori University, 86 Nishicho, Yonago, Tottori 783-8503 Japan; 26 University of Occupational and Environmental Health, 1-1, Iseigaoka, Yahatanishi-ku, Kitakyushu-shi, Fukuoka 807-8555 Japan; 27 Institute for Global Health Policy Research, Bureau of International Health Cooperation, National Center for Global Health and Medicine, Shinjyuku-ku, Toyama, 162-8655 Tokyo, Japan

**Keywords:** Cardiovascular disease, Mortality, Education level, Parity

## Abstract

**Background:**

While women’s parity status and education level have independent associations with cardiovascular and other diseases, no studies have evaluated the additive interaction of these two factors. Therefore, we examined the additive interaction between parity and education level on mortality from stroke, coronary heart disease, total cardiovascular disease, cancer, non-cardiovascular disease, and non-cancer causes, and all causes in Japanese women.

**Methods:**

This study followed 41,242 women aged 40–79 years without a history of cardiovascular disease or cancer from 1988 to 1990 until 2009. Baseline parity and education level were classified into four categories, with highly educated parous women as the reference group. Cox proportional hazards regression analyses were performed to calculate the risk of mortality. We also assessed the additive interactions between parity and education level on mortality from cardiovascular disease and other causes using the relative excess risk due to interaction obtained using Cox models.

**Results:**

During the median follow-up period of 19.1 years, we identified 6299 deaths. In a multivariable model adjusted for cardiovascular disease and other disease risk factors, nulliparous women with low education levels had increased multivariable-adjusted hazard ratios of 1.67 (95% confidence interval [CI] 1.13, 2.47) for stroke, 1.98 (95% CI 1.15, 3.39) for coronary heart disease, 1.71 (95% CI 1.34,2.18) for total cardiovascular disease, 1.69 (95% CI 1.33, 2.14) for non-cardiovascular and non-cancer, and 1.51 (95% CI 1.30, 1.75) for all-cause mortality when compared with highly educated parous women. Moreover, we observed significant additive interactions between parity and education level on total cardiovascular disease mortality (P = 0.04), non-cardiovascular disease and non-cancer mortality (P = 0.01), and all-cause mortality (P = 0.005).

**Conclusions:**

Nulliparity and low education levels are super-additively associated with total cardiovascular disease, non-cardiovascular and non-cancer, and all-cause mortality risks, suggesting that nulliparous women with low education levels need specific support for preventing mortality related to cardiovascular and other diseases.

**Supplementary Information:**

The online version contains supplementary material available at 10.1186/s12905-022-01805-y.

## Background

Education level is a key indicator and the most used measure of socioeconomic status (SES). Moreover, it is a crucial factor that affects the risk of cardiovascular disease (CVD) mortality, as well as health in later life [1–5]. Several studies have revealed worldwide associations between low education levels and increased risk of CVD mortality [6–12]. For example, longitudinal studies on mortality conducted in eight European countries, which included 51 million person-years, have revealed that CVD deaths account for 60% of the difference in total mortality between women with a low education level and those with a high education level [10]. Previous studies conducted in Japan have revealed that participants with a low education level had a 1.2- to 2.0-fold higher risk of CVD mortality than did participants with a high education level [7–9].

Other reports have described the associations between parity and CVD mortality [13–15]. The Israel Longitudinal Mortality Study II of 62,822 participants aged 65–89 years, who were followed up for 9 years, found that nulliparous women had a 2.4-fold higher risk of CVD mortality [16]. The American Cancer Society Cancer Prevention Survey II cohort study (585,445 participants) revealed that nulliparous women had a 1.6 to 2.4-fold higher risk of CVD mortality than parous women [15]. SES has been recognized as one of the determinants of health [3, 5, 6]. As reported previously, there is an inverse association between SES and all-cause, CVD, and cancer mortality [5, 17]. On the other hand, several studies have reported that parity is one of the risk factors for CVD and other causes [13–16]. However, few studies have examined the additive effects of parity and SES on CVD mortality and other causes.

It is important to utilize additive interaction for this research question because derivations from additivity are more important from a public health standpoint. In this context, two risk factors are independent when the number of events is also independent of whether they act jointly or not [18]. In this study, we hypothesized that both nulliparity and low education level might have more than additive interactions, resulting in a higher risk of mortality associated with CVD and other causes. Thus, this large cohort study aimed to evaluate the additive interaction of parity and education level on mortality associated with stroke, coronary heart disease (CHD), total CVD, cancer, non-CVD and non-cancer causes, and all causes among Japanese women.

## Methods

### Study population

The Japan Collaborative Cohort (JACC) study, sponsored by the Ministry of Education, Culture, Sports, Science, and Technology of Japan, is a nationwide multicenter collaborative study designed to prospectively evaluate various risk factors and protective factors for cancer incidence and mortality. The details of the methodology used in this study have been described elsewhere [19]. Briefly, a baseline survey was conducted from 1988 to 1990. In total, 110,585 participants (46,395 men and 64,190 women), aged 40–79 years, and residing in 45 areas in seven of eight districts in Japan, completed self-administered questionnaires. In our study, we enrolled residents living in 22 target areas. In 20 areas, enrollees were those who had undergone a basic health examination that was conducted in accordance with the Health and Medical Service Law for the Aged; in two areas, enrollees were identified based on a health checkup for atomic bomb survivors.

Among the 64,190 women who participated in the study, 13,345 were excluded because they did not answer questions regarding parity and/or education level on the questionnaire. In addition, 7330 women were excluded because they did not provide complete information regarding parity and/or education level. Furthermore, 2273 women were excluded due to a history of stroke, CHD, or cancer. Therefore, 41,242 women were included in the analysis. There were no substantial differences in risk factors for CVD and other diseases between individuals with and without complete information regarding parity and/or education level.

### Mortality surveillance

Follow-up surveys were conducted every 1–2 years to determine participant survival. The causes and dates of death were identified by reviewing all death certificates from each area. According to the 10^th^ revision of the International Classification of Diseases (ICD), mortality was classified as being due to stroke (I60–I69), CHD (I20–I25), total CVD (I01–I99), cancer (C00–C97), and non-CVD and non-cancer causes (all other ICD-10 codes). These outcomes were monitored until December 31, 2009, except in several areas where follow-up was terminated at the end of 1999 (6 areas), 2003 (5 areas), or 2008 (2 areas). The median follow-up period was 19.1 years (interquartile range 11.2–20.6 years).

### Data collection

Participants’ baseline characteristics were obtained using self-administered questionnaires that evaluated medical history and lifestyle-related items, such as diet, physical activity, drinking, smoking, and education level. For women, the questionnaires also evaluated parity status, age at menopause, and history of sex hormone use.

### Categories of parity status

Reproductive history was assessed using the following self-reported question: “How many times did you experience delivery?” We defined the number provided in the answer as the parity number. We did not further check with the women whether they understood the meaning of the question. The parity number given could then be considered to include the number of stillbirths and/or miscarriages. Based on the response to this question, women with a parity number of 0 were classified as nulliparous women (nulliparity), and those with a parity number of ≥ 1 were classified as parous women (parity).

### Categories of education level

Education level was assessed using a single question: “How old were you when you last attended school?” For the present study, the responses were classified as low education level (≤ 15 years old) and high education level (> 16 years old).

### Statistical analyses

Parity and education level were categorized as nulliparous women with a low education level, parous women with a low education level, nulliparous women with a high education level, and parous women with a high education level (the reference group). Based on these categories, we calculated the mean age, age-adjusted mean, and prevalence of CVD and other diseases of covariance for the mean values and a logistic regression model for the prevalence. Cox proportional hazards regression analyses were then performed to calculate the age-adjusted and multivariable-adjusted hazard ratios (HRs) and 95% confidence intervals (95% CIs) to determine the associations between the four categories and mortality associated with stroke, CHD, total CVD, cancer, non-CVD and non-cancer causes, and all-causes. All covariates were chosen based on prior research [3–5, 11, 20, 21], and were adjusted at the same time. We constructed multivariable hazard models adjusted for baseline age (years), body mass index (BMI) (kg/m^2^), history of sex hormone use (yes or no), weekly hours of habitual exercise and/or walking (almost never, 1–2 h, 3–4 h, and ≥ 5 h), daily hours of exercise and/or walking (almost never, < 0.5 h, 0.6–0.9 h, and ≥ 1 h), smoking status (never, ex-smoker, current smoker [1–19 cigarettes/day], and current smoker [≥ 20 cigarettes/day]), alcohol consumption (non-drinker, ex-drinker, current drinker [0.1–22.9 g/day, 23.0–45.9 g/day, 46.0–68.9 g/day, and ≥ 69.0 g/day]), daily duration of sleep (< 4.5 h, 4.5–5.4 h, 5.5–6.4 h, 6.5–7.4 h, 7.5–8.4 h, 8.5–9.4 h, and ≥ 9.5 h), level of perceived mental stress (high, medium, or low), baseline history of hypertension (yes or no), baseline history of diabetes (yes or no), and regular employment at baseline (yes or no). Moreover, we performed sensitivity analysis to examine the effect of the difference in the parity number on CVD and other causes. The parity status was categorized into nulliparity, moderate parity (1, 2, and 3) and high parity ≥ 4. The three parity statuses and two education levels (high and low) were further classified into six categories: (1) nulliparous women with a low education level, (2) nulliparous women with a high education level, (3) moderate parous women with a low education level, (4) moderate parous women with a high education level (the reference group), (5) high parous women with a low education level, and (6) high parous women with a high education level. The additive interactions between parity and education level for CVD and other causes of mortality were estimated using the relative excess risk due to interaction (RERI) obtained using Cox hazard models. RERI > 0 indicates the additive interaction of nulliparity and low education. RERI reflects the excess risk due to the interaction of nulliparity and low education relative to the risk of parity combined with a high educational level. We calculated RERI and that of the 95% confidence interval and P value for CVD and other mortality using the delta method (see Additional file 1) [22]. In addition, we tested the age-adjusted rates for CVD mortality, non-CVD and non-cancer mortality, and all-cause mortality according to parity and education level using the direct standardization method; this was based on deaths per 1000 individuals per year, and the age distribution from the national model population in 1985. All analyses were performed using SAS software (version 9.4; SAS Institute Inc., Cary, NC, USA), and the differences were considered statistically significant at a two-tailed P-value of < 0.05.

## Results

During the follow-up period, we identified 903 deaths from stroke, 386 from CHD, 2081 from total CVD, 2002 from cancer, 2216 from non-CVD and non-cancer causes, and 6299 from all causes. A total of 2420 participants have been censored for loss to follow-up. The mean parity number was 2.7, with a range of 0–13. Based on the original parity classification, 3.8% of the women were considered nulliparous. The mean age at the last school attendance was 16.7 years (range 6–58 years), with 61.0% of women having a high education level. The proportion of education levels and parity were as follows: 58.5%, parous women with high education levels; 2.4%, nulliparous women with high education levels; 37.7%, parous women with low education levels; and 1.4%, nulliparous women with low education levels.

Table 1 shows the mean ages, age-adjusted means, and prevalence of CVD and other disease risk factors according to parity and education level. Relative to parous women with a high education level, nulliparous women with a low education level were more likely to be older and have a higher BMI. In addition, nulliparous women with a low education level were more likely to have a history of hypertension and less likely to be married.

**Table 1:** Age-adjusted prevalence of CVD risk factors according to parity and education level

	Low education	Low education	High education	High education
	Nulliparous	Parity ≥ 1	Nulliparous	Parity ≥ 1
*No. at risk* (%)	565 (1.4)	15,528 (37.7)	994 (2.4)	24,155 (58.5)
Age, years	61.8	59.7	57.4	55.3
Body mass index, kg/m^2^	23.1	23.2	22.3	22.7
Married, %	69.4	85.1	68.6	86.7
Age at menopause, years	47.3	48.4	47.4	48.9
Current or previous hormonal drug use, %	8.2	3.7	12.1	5.1
Habitual exercise or walking, %	64.0	70.7	62.0	70.8
Current smoker, %	1.1	5.5	8.4	4.1
Current alcohol drinker, %	20.6	22.4	27.1	24.1
Sleep, h/day	7.3	7.1	7.1	7.0
History of hypertension, %	22.3	22.9	19.5	21.1
History of diabetes, %	4.3	3.8	4.1	3.7
High perceived mental stress, %	17.9	18.4	24.8	21.1
Regular employment, %	33.0	33.7	34.5	32.8

The HRs (95% CI) for mortality from CVD and other causes and RERI (95% CI) and P-value according to the four categories of parity and education level are shown in Table 2. Relative to parous women with a high education level, nulliparous women with a low education level had the highest age-adjusted mortality risks for total CVD, non-CVD and non-cancer causes, and all causes. These associations remained statistically significant after adjusting for the CVD and other disease risk factors. Significant additive interactions were observed for total CVD mortality , non-CVD and non-cancer mortality, and all-cause mortality (Table 2).

**Table 2:** Mortality from CVD and other causes and RERI according to education level and parity

	Education level				RERI (95%CI)	P value for RERI
	Low	Low	High	High		
	Parity					
	Nulliparous	Parity ≥ 1	Nulliparous	Parity ≥ 1		
Person-years	8686	246,667	16,318	400,450		
**Total cardiovascular disease**						
No	72	1093	55	861		
Age-adjusted HR (95%CI)	1.92 (1.51–2.44)	1.23 (1.12–1.35)	1.14 (0.87–1.50)	1.00	0.89 (0.16–1.61)	0.04
Multivariable HR (95%CI)	1.71 (1.34–2.18)	1.19 (1.08–1.30)	1.15 (0.87–1.52)	1.00		
Age-adjusted mortality rate	7.76	5.45	5.21	4.21		
**Cancer**						
No	35	873	56	1038		
Age-adjusted HR (95%CI)	1.05 (0.75–1.48)	1.03 (0.94–1.13)	1.12 (0.86–1.47)	1.00	− 0.04 (− 0.50 to 0.41)	NS
Multivariable HR (95%CI)	1.02 (0.73–1.44)	1.01 (0.92–1.11)	1.11 (0.85–1.46)	1.00		
Age-adjusted mortality rate	4.61	4.64	5.30	4.37		
**Non-CVD and non-cancer**						
No	76	1116	78	946		
Age-adjusted HR (95%CI)	1.88 (1.49–2.38)	1.17 (1.07–1.27)	1.48 (1.17–1.86)	1.00	1.13 (0.24–2.02)	0.01
Multivariable HR (95%CI)	1.69 (1.33–2.14)	1.12 (1.03–1.23)	1.47 (1.16–1.85)	1.00		
Age-adjusted mortality rate	8.81	5.65	7.11	4.58		
**All-cause**						
No	183	3082	189	2845		
Age-adjusted HR (95%CI)	1.64 (1.41–1.91)	1.14 (1.08–1.20)	1.25 (1.08–1.45)	1.00	0.65 (0.26–1.04)	0.005
Multivariable HR (95%CI)	1.51 (1.30–1.75)	1.10 (1.05–1.16)	1.25 (1.08–1.45)	1.00		
Age-adjusted mortality rate	21.18	15.74	17.61	13.16		

Multivariable adjustment: age, body mass index, history of hormonal drug use, habitual exercise or walking, current smoking, current drinking, sleep hours, history of hypertension, history of diabetes, perceived mental stress, and regular employment

RERI > 0 indicates additive interaction of nulliparity and low education

HR, hazard ratio; CI, confidence interval; CVD, cardiovascular disease; RERI, the relative excess risk due to interaction; NS, not significant

There were no associations of parity and education level with the risk of cancer mortality.

Table 3 shows the HRs (95% CI) for mortality from stroke, CHD, and RERI (95% CI) and P-value according to the four categories of parity and education level. Relative to parous women with a high education level, nulliparous women with a low education level had the highest age-adjusted mortality risks for stroke and CHD. These associations remained statistically significant after adjusting for the CVD risk factors. Significant additive interaction was not observed (Table 3).

**Table 3:** Mortality from stroke, CHD, and RERI, according to education level and parity

	Education level				RERI(95%CI)	P value for RERI
	Low	Low	High	High		
	Parity					
	Nulliparous	Parity ≥ 1	Nulliparous	Parity ≥ 1		
*Person-years*	8686	246,667	16,318	400,450		
**Stroke**					1.07 (− 0.30 to 2.44)	0.08
No	28	492	24	359		
Age-adjusted HR (95%CI)	1.83 (1.25–2.70)	1.36 (1.19–1.56)	1.22 (0.80–1.84)	1.00		
Multivariable HR (95%CI)	1.67 (1.13–2.47)	1.33 (1.15–1.53)	1.24 (0.82–1.88)	1.00		
Age-adjusted mortality rate	2.94	2.47	2.41	1.72		
**Coronary heart disease**					1.46 (− 0.81 to 3.73)	0.20
No	15	204	10	157		
Age-adjusted HR (95%CI)	2.17 (1.28–3.69)	1.24 (1.01–1.54)	1.14 (0.60–2.15)	1.00		
Multivariable HR (95%CI)	1.98 (1.15–3.39)	1.20 (0.97–1.49)	1.16 (0.61–2.21)	1.00		
Age-adjusted mortality rate	1.64	1.19	0.83	0.76		

Multivariable adjustment: age, body mass index, history of hormonal drug use, habitual exercise or walking, current smoking, current drinking, sleep hours, history of hypertension, history of diabetes, perceived mental stress, and regular employment

RERI > 0 indicates additive interaction of nulliparity and low education

HR, hazard ratio; CI, confidence interval; CHD, coronary heart disease; RERI, the relative excess risk due to interaction

The results of the sensitivity analysis were that relative to moderately parous women with a high education level, nulliparous women with a low education level had the highest age and multivariable-adjusted mortality risks for total CVD, non-CVD and non-cancer causes, all causes, stroke, and CHD. Relative to moderately parous women with a high education level, moderately parous women with a low education level, highly parous women with a low education level, and highly parous women with a high education level had increased multivariable-adjusted hazard ratios of 1.25 (95% CI 1.11, 1.41), 1.28 (95% CI 1.12, 1.46), and 1.18 (95% CI 1.02, 1.37) for total CVD, 1.10 (95% CI 1.03, 1.17), 1.22 (95% CI 1.13, 1.32), and 1.13 (95% CI 1.04, 1.323) for all causes, and 1.45 (95% CI 1.21, 1.75), 1.59 (95% CI 1.30, 1.94), and 1.42 (95% CI 1.13, 1.77) for stroke mortality.

## Discussion

To our knowledge, this large-scale study on 40–79-year-old Japanese women is the first study to reveal that parity and education level had super-additive associations for mortality from CVD, non-CVD and non-cancer, and all causes. From a public health perspective, deviations from the additive interaction of two dichotomous risk factors resulted in a multiplicative association which means a super-additive association [23, 24]. Therefore, in this study, the combination of nulliparity and low education level had super-additive associations for CVD and other causes of mortality. Previous studies have evaluated the independent association between CVD mortality and parity [13–16] or education level [6–12]. Our findings regarding the association between low education levels and a higher risk of CVD and other mortality are consistent with those of previous studies. For instance, Mackenbach et al. [11] conducted an international study that included the United States and 11 Western European countries; a low education level was associated with an elevated risk of CVD mortality in all 12 countries. In addition, a large prospective cohort study of 303,036 Asians and Australians indicated that the risks of CVD mortality were 2.5- and 1.2-fold higher among Asians and Australians with low education levels, respectively [25].

We also observed an association between nulliparity and a higher CVD mortality, which is also consistent with previous findings from Western countries and Japan [14, 26–28]. For example, a meta-analysis of 10 cohort studies in Western and Asian countries found that parous women had a 31% lower risk of CVD mortality than nulliparous women [14]. In addition, a prospective study of 39,870 Japanese women indicated that nulliparous women had a 1.6-fold higher risk of stroke mortality and a 1.3-fold higher risk of total CVD mortality than women with a single child [28]. While the biological mechanism involved in the association between nulliparity and higher CVD mortality remains unclear, nulliparity has been associated with higher estradiol levels, which may lead to elevated blood pressure [29] and increased blood vessel stiffness [30]. Moreover, nulliparous women with infertility issues often have polycystic ovary syndrome [31] or experience miscarriage [32] and pregnancy loss [33], which can also contribute to a higher CVD mortality risk.

Few studies have investigated the additive interaction of parity and education level on CVD mortality. One population-based cohort study of 527,964 Norwegians aged 40–69 years found that women with a parity number of ≥ 3 and low education level had a 2.6-fold higher risk of CVD mortality than women with a parity number of 2 and high education level [34]. However, this study did not examine the combined effects of nulliparity and low education level on CVD mortality. The present study revealed that the risks of stroke mortality, CHD mortality, and total CVD mortality were the highest among nulliparous women with a low education level, followed by parous women with a low education level. Several studies have suggested that good lifestyle behaviors (e.g., being physically active, having a moderate BMI, and not smoking) are associated with a reduced risk of CVD mortality; however, individuals with a low education level are more likely to have poor lifestyle behaviors [20, 21, 35, 36]. Similarly, the present study revealed that women with a low education level were more likely to have a higher mean BMI and a history of hypertension, both of which are risk factors for CVD. The lack of association between cancer mortality and both parity and education level is not completely clear. One possible explanation is that parity and education level are related to lifestyle factors and psychosocial factors and do not have much of an association with cancer mortality compared with CVD mortality. Meanwhile, the highest risk of non-CVD and non-cancer mortality was observed among nulliparous women with a low education level, followed by nulliparous women with a high education level. We calculated the specific causes of non-CVD and non-cancer mortality and found that the most common causes were pneumonia (39.2%), injury or suicide (26.3%), and natural death (11.1%). Moreover, relative to parous women, nulliparous women (regardless of education status) had higher age-adjusted annual mortality rates for pneumonia (3.94 vs. 6.18) and injury or suicide (2.77 vs. 3.42). One conceivable reason for this may be that the age-adjusted mortality rate of injury/suicide among nulliparous women was higher than that among parous women; this is supported by prior studies [37, 38]. Moreover, the age-adjusted mortality rate of pneumonia, one of the leading causes of all-cause mortality, was higher among nulliparous women compared with parous women in this study. These factors seemed to strongly affect mortality due to non-CVD and non-cancer causes, as well as all causes, among nulliparous women.

Psychosocial mechanisms for the super-additive association of nulliparity and low education on CVD and other disease mortality have not been clarified. According to previous studies, one conceivable reason is that parous women, regardless of their education status, might have been reproductively healthy, had social networks or supporters, and developed positive health-related behaviors due to childrearing [39–41]. Furthermore, women with a high education level, regardless of their parity status, are more likely to have or be able to access health-related information, better treatment, or greater health knowledge, which may explain the association with a reduced CVD risk and other mortality causes [42–45]. Thus, women with a combination of nulliparity and low education level might be vulnerable to losing the opportunity or motivation to practice health-related behaviors and acquire social support, which may help attenuate their CVD risk and other mortality causes. As a result, the combination of nulliparity and low education level has super-additive interactions, rather than additive interactions, on CVD and other disease mortality. These results suggest that nulliparous women with a low education level require extensive additional support to improve their lifestyle habits and promote behaviors that can help prevent CVD-related mortality and other diseases.

This study has several strengths. First, the participants were selected from a large-scale prospective cohort that included participants throughout Japan and a total of 2081 CVD deaths. The prospective design may have increased the robustness of our findings by eliminating selection and recall bias. Second, the original study collected baseline data for numerous factors, which allowed us to adjust the analyses for potentially confounding variables such as CVD risk factors and psychological factors. Third, we used education level as a measure of SES, which is one of the most powerful factors affecting the health of individuals [46, 47].

Our study has several limitations. First, we did not have access to data regarding clinical CVD risk factors, such as elevated blood pressure, blood glucose, or lipid values. Thus, we adjusted the analyses using histories of hypertension and diabetes mellitus for accounting for these factors. Second, selection bias was possible, as we excluded 18.9% of participants for incomplete information regarding parity, education levels, or medical history. Compared to the participants enrolled in this study, the excluded participants were older; more likely to be current smokers and have a history of hypertension; and less likely to perform habitual exercises, such as walking. However, the proportions of other baseline characteristics were similar. Third, the nulliparous women enrolled in the present study may not be representative of nulliparous women in current settings, as the Economic and Social Research Institute in Japan has reported that the lifetime non-marriage rate has increased from 4.5% in 1980 to 14.1% currently. Unmarried and/or childless women in the 1980s might have had a low SES or problems with physical or mental conditions compared with Japanese women in the present, who may voluntarily choose not to marry and/or have children. Thus, the additive interaction of nulliparity and low education level with CVD mortality might be smaller in a more recent cohort. Fourth, we did not obtain detailed information regarding parity status (e.g., abortion, stillbirths), and did not ensure that the women understood the question. This may have led to an underestimation of nulliparity, thereby reducing the real associations that might have been stronger. Finally, highly parous (≥ 4) women may have a higher risk of CVD mortality than moderately parous (1–3) women based on the results from this study and previous study [4, 5, 14].However, the results of the sensitivity analysis of the different parity classifications on the CVD risk of parity and educational history was little difference compared to the original results. As for mortality from total CVD, all causes, and stroke, highly parous women, regardless of their education level, were higher hazard ratios compared to moderately parous women. On the other hand, there was no difference in the effect of moderate parity and high parity on non-CVD and non-cancer and CHD risk of mortality. Therefore, we adopted nulliparous or parous as additive dichotomized variables generally easy to understand.

## Conclusions

The present study revealed that nulliparous women with a low education level had an increased risk of mortality due to stroke, CHD, total CVD, non-CVD and non-cancer causes, and all causes. Moreover, nulliparity and a low education level had super-additive associations for the risk of total CVD, non-CVD and non-cancer causes, and all-cause mortality. These results suggest that nulliparous women with a low education level require extensive additional support to promote behaviors that can help prevent mortality related to CVD and other diseases.

## Supplementary Information

**Additional file 1**. Calculations for relative excess risk due to interaction (RERI) and the 95% confidence interval for stroke mortality.

## Data Availability

The datasets used and/or analyzed during the current study are available from the corresponding author on reasonable request.

## Abbreviations

BMI: Body mass index
CHD: Coronary heart disease
CI: Confidence intervals
CVD: Cardiovascular disease
HR: Hazard ratio
ICD: International Classification of Diseases
JACC Study: Japan Collaborative Cohort Study
RERI: Relative excess risk due to interaction
SES: Socioeconomic status
