# Supplementary material for: Super-additive associations between parity and education level on mortality from cardiovascular disease and other causes: the Japan Collaborative Cohort Study
Source: BMC Womens Health. 2022 Jul 6;22:278. doi: 10.1186/s12905-022-01805-y (PMC9261019; doi:10.1186/s12905-022-01805-y)
Supplement: Supplementary file 1 — Additional file 1. Calculations for relative excess risk due to interaction (RERI) and the 95% confidence interval for stroke mortality. [file 12905_2022_1805_MOESM1_ESM.docx]

**Additional file 1**

The calculations for relative excess risk due to interaction (RERI) and the 95% confidence interval for stroke mortality are shown below:

RERI = HR_11_ – HR_10_ – HR_01_ + 1,

= $e^{\beta1+\beta2+\beta3}- e^{\beta1}- e^{\beta2}+1$

= 2.50 – 1.11 – 1.32 ＋ 1

= 1.07

HR_ij_ is the adjusted hazard ratio relative to the reference. HR_11_, HR_10,_ and HR_01_ reflect the hazard ratios for nulliparous women with low education, nulliparous women with high education, and parous women with low education, respectively.

VAR (RERI) =$a_{1}^{2}$ × Var$\beta_{1}$ ＋ $a_{2}^{2}$ × Var$\beta_{2}+$　$a_{3}^{2}$ × Var$\beta_{3}$　+

2 ($a_{1}a_{2}$ × Cov ($\beta_{1} \beta_{2})+ a_{1}a_{3}$ × Cov ($\beta_{1} \beta_{3})+ a_{2}a_{3}$ × Cov ($\beta_{2} \beta_{3})$),

$a_{1}$ =　$e^{\beta1+\beta2+\beta3}- e^{\beta1}$

= 2.50 – 1.11

= 1.39,

$a_{2}$ = $e^{\beta1+\beta2+\beta3}- e^{\beta2}$

= 2.50 – 1.32

= 1.18,

$a_{3}$ =　$e^{\beta1+\beta2+\beta3}$

= 2.50

VAR (RERI) = 0.49, and SE (RERI) = 0.7. Hence, t = RERI / $\sqrt{VAR(RERI)}$ = 1.07/0.70 = 1.53, P = 0.08.

The 95% confidence interval RERI was estimated as follows:

RERI ± 1.96 × SE (RERI) = (–0.30, 2.44)
